# COVID-19 pandemic and dental practice in Ghana

**DOI:** 10.4314/gmj.v54i4s.15

**Published:** 2020-12

**Authors:** Sandra A Hewlett, Paa Kwesi Blankson, Akua B Konadu, Kofi Osei-Tutu, Dennis Aprese, Maxwell Adjei, Alfred E Yawson, Peter Donkor, Ebenezer A Nyako

**Affiliations:** 1 Department of Restorative Dentistry, University of Ghana Dental School, University of Ghana, Accra, Ghana; 2 Department of Oral and Maxillofacial Surgery, Korle-Bu Teaching Hospital, Accra, Ghana; 3 Dental Department, Effia-Nkwanta Regional Hospital, Ghana Health Service, Takoradi, Ghana; 4 Dental Department, Greater Accra Regional Hospital, Ghana Health Service, Accra, Ghana; 5 Department of Community Health, University of Ghana Medical School, University of Ghana, Accra, Ghana; 6 Department of Surgery, Kwame Nkrumah University of Science and Technology, Kumasi, Ghana; 7 Department of Surgery, School of Medicine and Dentistry, Kwame Nkrumah University of Science and Technology, Kumasi, Ghana

**Keywords:** COVID-19, Pandemic, Dentistry, Ghana, Dental Practice

## Abstract

With the advent of the COVID-19 pandemic, healthcare systems and their provision of care has globally been challenged, including the delivery of Oral healthcare. In Ghana, it has become imperative that healthcare delivery including the practice of Dentistry and its sub-specialties be re-oriented in our peculiar setting to ensure minimal risk of spread of the infection. This article discusses the impact of COVID-19 on the practice of Dentistry in the country.

## Introduction

### Covid-19 and its emergence in Ghana

At the end of 2019, the world was hit with a major public health threat, the COVID-19 disease, which has affected millions of people globally^1^, and tested healthcare systems and provision, including Oral health. While coping strategies may depend on how robust each healthcare delivery system is, that of Ghana and sub-Saharan Africa (SSA) in general, may require a critical appraisal and positioning.

The state of Oral health in Ghana is quite disparate, with varying disease burden across different populations.^2^ While some communities have minimal disease, others have relatively higher prevalence of dental caries, periodontal disease and other Oral health disorders. Oral health is therefore essential to the healthcare system of Ghana, and critical to our achievement of Universal Health Coverage (UHC).

Since the World Health Organization (WHO) declared the virus as a pandemic in March 11th, there have been tremendous and conscientious efforts to mitigate it's spread. These include intensified disease surveillance, prevention of community spread, development of serological tests, as well as the initiation of vaccine development, treatment modalities and continuous research.^4^ Ghana recorded its first two confirmed cases on 12^th^ March 2020. Since then, the number of confirmed cases have since risen exponentially, forcing many local reactions, and challenging the healthcare system at several fronts including the practice of Dentistry.

### Impact on Dental Health

As with most specialist care and clinics, there seems to have been a phase of uncertainty, while further understanding of the virus is being sought. As a result of fear of contracting the virus, or as a moral duty to prevent spreading COVID-19 among their patients, Dental professionals have limited their services.^6^ In China, while patient attendance significantly reduced, there was an interesting change in the pattern of dental problems. The number of dental and oral infections increased from 51% of pre-COVID-19 to 71.9% during COVID-19, and dental trauma decreased from 14.2% to 10.5%.^7^ Anecdotal evidence from the Korle-Bu Teaching Hospital, Ghana, revealed a similar observation with an upsurge in severe dental-related admissions during this period, and a record of associated mortalities. This could have been due to reduced routine utilization of oral healthcare services during the pandemic.

From the perspective of practitioners, there has been reasonable concern about the financial implications of the pandemic.^6^ Though some practitioners have been working, there seems to have been a general inclination to suspend elective care and focus on emergencies, which do not generate as much revenue. This, as well as the cost of procuring enhanced PPE's has had a huge toll on income generated by most Dental clinics and brings into question their sustainability if the pandemic lingers for a long time.

### Peculiar risk to the Oral health worker

Substantiated features of COVID-19 include evidence of the transmissibility of the virus during close contact through respiratory droplets and by fomites.^8^ Thus the virus can spread directly from person to person when a COVID-19 infected person coughs or exhales producing droplets that reach the nose, mouth or eyes of another person.^9^ Furthermore, transmission through smaller droplet nuclei that propagate through air at distances longer than one meter is possible with aerosol generating procedures during dental clinical care of COVID-19 patients, aided by air-driven equipment used in the practice of dentistry.^8^ Furthermore, the viral load in saliva has been reported to be very high in both symptomatic and asymptomatic patients. These factors, although not definitely confirmed, are putatively felt to put both the Oral health worker and the patient at higher risk of infection, as procedures are carried out in close proximity to patients.^10^

### Response of the Dental fraternity

It is quite evident that the emergence of the pandemic has drawn meaningful responses from many citizens to assist in their own ways in the fight against COVID-19. The Dental fraternity was not left out in volunteering and redeployment of its members with some institutional assignments, as well as social responsibilities.^6^ In Ghana, Dental surgeons have commendably been part of several COVID-19 response units for different hospitals, and the Ministry of Health's contact tracing teams. At the international and local community level, direction has been provided by the World Health Organisation (WHO)^11^, the American Dental Association (ADA)^12^, the Centers for Disease Control and Prevention (CDC)^13^, World Dental Federation^14^, Ministry of Health/Ghana Health Service, Ghana Medical Association, and the Ghana Dental Association. The greater risk to the Dental Surgeon however demands more efforts by stakeholders to ensure that Oral health workers are motivated and equipped to work without unwarranted risk or harm.

### Recommendations

The teaching and practice of Dentistry in Ghana has been informed by existing guidelines such as the standard precautions^15^, the Universal, and transmission-based precautions^16^. This is a good time for the Dental profession to revise and enforce these guidelines, bearing in mind that the unique nature of Dental procedures, instrumentation and patient care settings require specific strategies directed at the prevention of transmission of diseases among Dental health care workers and their patients.

With the advent of COVID-19, Government Agencies, International, and Non-Governmental Organizations, have made several recommendations, which are beneficial to the practice of Dentistry. Informed by prevailing evidence and other guidelines^17^,^5^,^18^, we make further recommendations for all Dental surgeons and settings in Ghana.

### Recommendations for Dental practice in Ghana Category and Recommendation

Dental OPDs and Administration

Recommendation

- Until more sensitive screening tools are made available, all Dental clinics and settings should have screening posts for all patients where an evaluating questionnaire is filled, and infrared thermometer is used to take temperature taken.
- Screening/triage officers should wear appropriate PPEs and maintain all existing protocols.
- All patients and staff must wash and disinfect their hands before entering the waiting area.
- Seating in OPD and waiting areas must observe social/physical distancing protocols of a minimum of 1.5 meters apart.
- Streamline patient and staff traffic within facilities eg. dedicated entrance and exit points
- Strict booking systems for elective procedures to prevent crowding in waiting areas.
- Do not treat patients that have respiratory conditions and/or fever without appropriate testing
- All Oral health workers must be screened, and be in face masks at all times.
- Waiting areas, floors, tables, handles and other surfaces must be disinfected regularly, and all books and brochures removed.
- Type of useful disinfectants available in Ghana should be named to help practitioners choose effectively and avoid confusion.

Treatment area

Recommendation

- Standard cross-infection guidelines must be strictly adhered to
- All patients should sanitize their hands again before entering the treatment area and before sitting in the Dental chair
- As much as possible, patients should enter treatment area alone, without company
- All operating Dental surgeons and assistants must be fully protected. This includes N-95 mask, face shield, gown, boots and hair protection.
- The use of single-use scrubs for Dental surgeons and assistants is recommended for this period
- There should be demarcated donning-on and doffingoff areas in the clinics. For reusable gowns, there should be a well-structured sterilizing system.
- Before treatment, patients should be asked to rinse and gargle with diluted H2O2 1.5% for 1 minute and/**or 2**% chlorhexidine for 2 minutes.
- Do not compromise on the use of rubber dams, **i.e.**, as much as possible use rubber dam when doing restorative procedures
- Before cutting of access cavities, disinfect tooth with hydrogen peroxide solution.
- Despite all measures and protocols, minimize aerosol-producing procedures to the barest minimum **i.e.**, use of ultrasonic scalers, air polishing, air/water syringe, tooth prep with air turbine or air abrasion.
- Dental chairs and treatment areas must be disinfected after every patient

Admissions and in-patient care for Oral and maxillofacial conditions

Recommendation

- Provision should be made for in-patient isolation care for patients with high index of suspicion *Public health and human resource measures* Recommendation
- General Dental education should include topics on COVID-19
- Orderlies, cleaners and anyone with similar responsibilities should be particularly educated, equipped and motivated for more frequent and thorough discharge of duties.
- We recommend good collaboration between private and public services, to reduce crowding and allow for ease of referrals of high-risk patients.
- Dental departments and private clinics should have protocols on testing and quarantine/isolation procedures, for staff who will be exposed based on national guidelines
- The Ghana Dental Association should provide a help desk to assist with members who could be exposed, to facilitate collaborations, to provide general information, and to link members and allied workers to other clinicians and mental health experts as necessary.
- The Ghana Dental Association with the Ministry of Health should collaborate and provide frequent or monthly upgrades on changing preventive and treatment protocols during the pandemic period.

## Conclusion

With increasing and changing evidence regarding the highly infectious nature of COVID-19, it is important that the Ghanaian Dental surgeon and Oral Health worker adapt and stay updated. Personal protection should not be compromised, as it is proven that asymptomatic patients might well be infectious. Extreme precautions should be taken to prevent disease transmission
